# Social engineering in the concept of rational and irrational consumer behavior

**DOI:** 10.3389/fnut.2022.961929

**Published:** 2022-10-25

**Authors:** Lyubov Krestyanpol

**Affiliations:** Department of Applied Linguistics, Faculty of Foreign Philology, Lesya Ukrainka East European National University, Lutsk, Ukraine

**Keywords:** consumer behavior, rational consumption, smart packaging, web form, social engineering

## Abstract

In this work, the author researching the process of consumer behavior formation, and considers the possibility of its correction using social engineering methods. The author considers consumer behavior as a separate mandatory element of the product quality monitoring system in the concept of smart consumption. Consumer behavior as a complex of actions and reactions of a public entity in the field of consumption has been considered, assumptions of the economic component of consumer behavior have been formulated, and recommendations for consideration of consumer behavior in the context of social relations have been given. The formation of consumer behavior from the point of view of psychology has been described and considered certain factors that influence the formation of a certain type of consumer behavior. The author has developed strategies for influencing consumer behavior based on social engineering methods. Two approaches to applying strategies to rational and irrational behavior are proposed. The developed strategies will encourage the consumer to participate in the information system of data collection to monitor product quality. The author conducted research, the purpose of which is to determine the target audience that will participate in the process of informing about the product quality monitoring system. As a result of the research, a focus group was formed, which took part in an experiment with the use of strategies to influence consumer behavior.

## Introduction

Consumer behavior is a set of actions and reactions of the social subject in the field of consumption, which includes economic interest and social interaction. The economic component of such behavior involves: choosing the most profitable alternatives; the rationality of the acting subject, the presence of thinking about the results of behavior in his actions in terms of its effectiveness; conditionality of economic motives to maximize material benefits; awareness of possible ways to meet their needs. But, on the other hand, it is important to consider consumer behavior in the context of social relations, as described by the model of “homo economicus,” which main characteristics are: the conditionality of behavior by socio-cultural conditions; the impossibility of developing a rational scheme of human behavior: individual human actions may be spontaneous and unpredictable (1). Based on this, consumer behavior can be divided into rational and irrational. But is it possible to influence and change consumer behavior? This article considers the possibility of using social engineering methods to change the rational and irrational behavior of consumers.

Today society is required to adhere to the rational consumption of both resources and raw materials and food. The problem of food waste growth is becoming global. Every year, tons of ready-made food and semi-finished products are transported from the refrigerator to the landfill. The situation with food losses during production is not better as well. If the production process is controlled by the manufacturer, then this process during the sale and consumption is almost uncontrollable. In the points of sale and shops, the appearance of food waste is monitored directly by employees, who may not always be able to find expired goods in time. Such goods are usually disposed of in food waste, or consumers can purchase such goods through their negligence. In turn, consumers at the stage of consumption of goods also generate a significant percentage of food waste. This is due to a number of reasons. One of them is the long shelf life of products. Too long shelf life often seems suspicious to consumers, so they try to get rid of a product at least a few days before it expires. Another reason for food waste is the inability to calculate consumption. Experiments (2) show that people do not know how to determine correctly how much food they can eat over a period of time. Buying junk food, or so-called “gastronomic shopaholism” is a problem that exists in real life. Quite often consumers buy food that they do not like or consider not very useful because of curiosity or other non-obvious motives.

Another reason for the appearance of food waste is a violation of storage rules. Very often food becomes unfit for consumption due to improper storage. Violation of the rules of commodity neighborhood, re-freezing, failure of the temperature regime, all leads to the inevitable loss of useful properties.

Irrational attitude to food leads to unpleasant and even dangerous consequences. Improper approach to eating and storing food leads to unplanned financial costs. You have to buy food more often. Too many resources are spent on food production, so throwing them into landfills is also a significant impact on the environment.

The result of the study of this topic is the author's concept of smart consumption, which is described in detail in (3). These studies present an analysis of a new information collection system aimed at improving the concept of “smart” packaging. The authors propose an innovative approach to understanding the concept of “smart” packaging as an integral part of modern technical solutions and social responsibility. The articles highlight the possibility of using RFID data transmission technology to maintain the performance of the information collection system. Procedures for encoding RFID tags and obtaining the information are clearly defined and illustrated by experimental data. The main advantage of the proposed model is the ability to use time ID-tags to monitor product expiration limits in real-time.

However, the technical side is not always able to cover and solve the task. Unfortunately, this system does not take into account defects, damages and signs of microbiological spoilage that occur directly in the sales hall. Products marked with RFID tags actually control the expiration date, which is determined by the date of manufacture and the date of final consumption. In practice, expiration dates are entered on the RFID tag, and as these dates approach, the tag begins to signal and send information about the expired product to the database. However, if the conditions of storage or product proximity are violated, the goods may have signs of deterioration that neither the administrator of the trading hall nor the RFID tag can detect. Usually, such signs are detected by visitors to the store during the inspection of the product, or already in the process of consumption. Unfortunately, in practice, after detecting a defect or signs of deterioration, consumers do not always notify the administrator of the trading hall, explaining this by a lack of time or a lack of feedback means. Thus, in order to increase the efficiency of the system, the author considers the possibility of including in the system of monitoring the quality of consumer products, as a full-fledged independent element of this system.

The main task of the consumer is to detect defects and inform about the type of defect through the developed mobile application. However, each individual visitor to the store and, as a result, a potential user of the developed system, from the point of view of marketing has its own consumer behavior. Depending on the type of consumer behavior, the probability of his participation in the system will vary. Consumer behavior is influenced by psychological, personal, socio-cultural factors, factors of situational influence. The author analyzes the psychological factors of influence, in particular, the formation of a motive by methods of social engineering. For the first time, the possibility of using social engineering methods to shape consumer behavior is considered. The work offers a scheme of phases of consumer choice adapted to the research problem, as well as a set of strategies for influencing consumers with different consumer behavior.

## Literature review

The fundamental bases for the interpretation of the essence of consumer needs are formed in the works of A. Maslow, R. Blackwell, W. James, Their efforts created a solid theoretical and methodological foundation for the study of this issue, solved many of its methodological and applied aspects. Prerequisites for the theory of rational choice arose in the middle of the XVIII—early XIX centuries in the doctrines of the morality of the Scottish school of morality, whose representatives first proposed an individualistic concept of rational human behavior and drew attention to its fruitfulness to explain other social phenomena. A significant contribution to the development of the evolutionary theory of individual behavior was made by R. Nelson and S. Winter (4). Among the researches on the theory of consumer behavior, the work of Saeed (5) and Michael S. Miller attracts our attention (6).

The theory of rational consumption, the foundations of which were laid by R., Stark (7), Amartya Sen (8), J., Meyer, (9) and others in the development of “rational consumer budget” considers the possibility of determining targets for consumption. Physiological needs, functional household processes, social requirements can act as such. Rationality in economic theory is realized primarily in the model of “economic man.” “Economic man” is meaningfully defined as a set of relations of human exchange with the natural and social environment because this exchange provides him with the means to meet material needs (10). In her work O. Aleinikova (1) highlights the following features of an “Economic Man”:

- actions are subject to one motive—selfish pursuit of self-interest, which is expressed in maximized utility;
- identified needs that are limited only by the availability of resources;
- Rationality of decisions made;
- Autonomy in decision-making.

Neoclassicists divide consumer behavior into rational and irrational. The rational one includes:

- Target function (purposefulness of activity—a person seeks to best meet their needs, which means material needs, those that are met by external sources);
- External information available for selection and decision-making;
- Human intellectual capabilities: memory, which stores information about the hierarchy of needs, the degree of their satisfaction, and the mind, which enables to calculate the results of their possible actions, weigh their importance and choose the best option.

Irrational consumer behavior is based on logically unmotivated actions. The consumer performs certain actions unconsciously or under the influence of certain emotions. Modern marketing and advertising have sophisticated methods of persuasion in their arsenal, which use the latest advances in psychology, sociology and other sciences that study human behavior. Studying and taking into account irrational forms of decision-making is the gold mine of modern marketing, especially advertising. Marketing technologies use the whole possible arsenal of means to influence the consumer, based on the mechanisms of imitation, infection and suggestion (hypnosis, suggestion). Incentives for unconscious action are often hidden behind outwardly invulnerable forms of marketing influence, which the consumer can rationally analyze as may seem at first glance.

**Figure 1:**
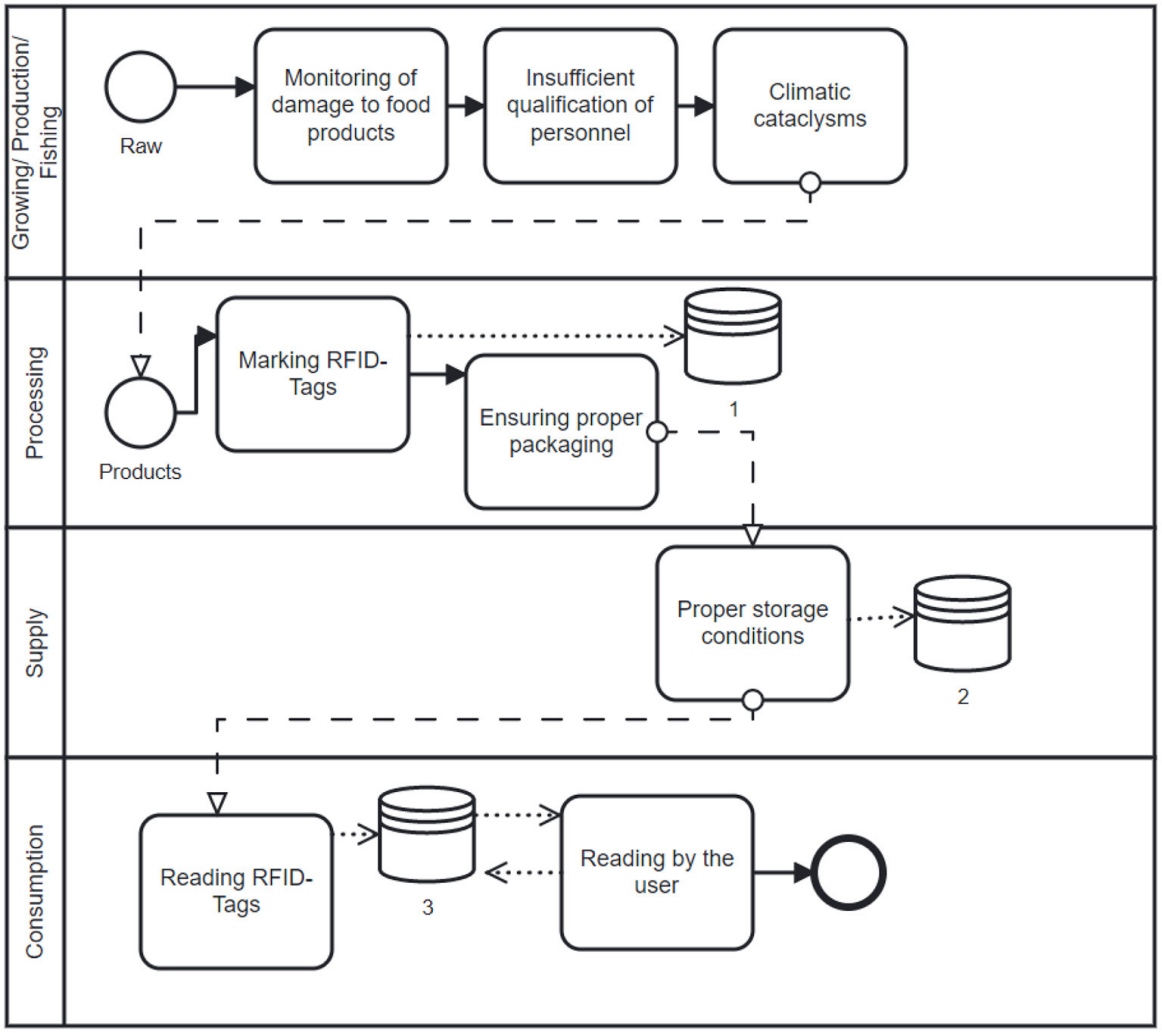
The process of monitoring product expiration limits. Source: Authors' depiction.

If we describe these two types of behavior in one word, then for the rational one the corresponding word is thought, and for the irrational one, it is feeling. An interesting example of the formation of consumer behavior is given in the works (11), where the author considers the formation of attitudes and intentions based on thoughts and feelings in Figure 1.

If we analyze the factors that influence the formation of consumer behavior, psychological factors are undoubtedly fundamental. Thoughts and feelings form an intention (motive), which accordingly creates consumer behavior. By influencing thoughts and feelings, you can adjust consumer behavior and cause the necessary actions in consumers. Accordingly, strategies for influencing the opinion and feelings of the respondent differ.

Personal factors include gender, age, family status, education, profession. In the study, these factors are also taken into account, because the author tries to determine the target audience that will actively participate in the work of the monitoring system, and also examines the dependence of the type of consumer behavior on age and gender.

Situational influence factors are considered in the context of the atmosphere in the store and the actions of other customers. Performance of the required actions by the buyer may cause incentives for other participants to perform these actions.

**Figure 2:**
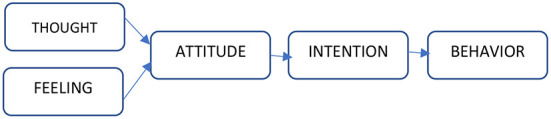
Formation of consumer behavior. Source: Researchers' elaboration based on research data.

All components of consumer behavior shown in Figure 2 are important elements that can be considered as a vector of attack in shaping the strategy of social engineering. The author considers the possibility of using social engineering methods to change consumer behavior.

The term “social engineering” is most often used in the context of fraud, cybercrime and manipulation. Today, there are many methods of using social engineering based on the manipulation of human fears, interests or trust. In his work, R. Moseley (12) considers various psychological models and the psychology of manipulating an individual or a group of people. However, social engineering can be used for the benefit of society to solve certain problems. This article is devoted to the use of social engineering methods to implement a policy of smart resources consumption.

## Methods and materials

The history of consumer behavior research is largely intertwined with the history of marketing thought (Sheth 1985), and thus each marketing era has had an effect onconsumer behavior research. In the early years of the development of the discipline, consumer behavior research methods focused on sampling, collecting data, andanalytical techniques (Clow and James 2013). The primary goal of marketing research at that time was to measure phenomena and consumer characteristics. Researchers also focused on measuring opinions, perceptions, preferences, attitudes, personalities, and lifestyles (13).

Today, new forms of data (e.g., big data, the Internet, social media) have become available, giving rise to the study of phenomena related to the relationship between firms and consumers.

This consumer behavior research project follows the following steps: research objectives; research design; sampling plan; data collection; data analysis; and reporting. The goals of the study are:

- determining the dependence of the type of consumer behavior on age and gender;
- formation of the necessary consumer behavior by methods of social engineering.

**Figure 3:**
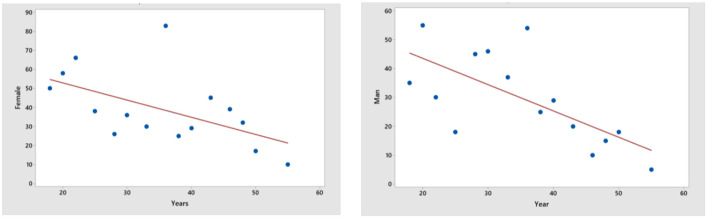
Distribution of respondents by age and gender. Source: Researchers' elaboration based on research data.

Different methods were used in the research, depending on the goal. The use of selective observation with the use of specially developed questionnaires—to form a sample population of the study and identify key characteristics and motives of consumer behavior. Quantitative survey methods were used to get a general idea of the feasibility of the project and to establish the target audience. The survey was conducted by filling out online forms. 1000 respondents took part in it. This survey made it possible to form a target audience by gender and age group. The results of the survey by gender and age are shown in Figure 3.

Qualitative methods of focus group research and experiment were used to study behavior.

Out of 1,000 respondents who completed the survey, focus groups were formed according to the age category of 20–30 and 35–40 years and gender: men, women. Table 1 shows the results of the focus groups. In Table 1, points 5, 6, 7 were formed based on the results of the focus groups. As a result of the research, it was possible to formulate types of strategies for the formation of certain consumer behavior for different gender and age groups.

**Table 1:** The results of the survey.

**No**	**Question**	**Yes**	**No**
1	Are you worried about the pollution of the environment with food waste?	87,5 %	12,5%
2	Would you like to allocate financial costs for food rationally?	100%	0
3	When buying food in the store, are you interested in the expiry date of the product?	81,2%	18,8%
4	There is an opportunity for the consumer to detect and point out defects in the goods in the trading hall of the store. Will you detect and point out defects?	76%	27%
5	There is an opportunity for the consumer to detect and point out defects in the goods in the trading hall of the store. Choose the way that will be convenient for you to do it.	50% using a mobile application on your phone;	
		25% with the help of special equipment in the store;	
		25% calling the administrator	
6	When buying food in the store you find (fault, contamination, spoilage of the product). Your actions?	62, 5% are worried about the defect, but will not call the administrator, because it will take a long time;	
		31.3% will inform the administrator about the decline in product quality;	
		6.3% leave	
7	The store has a system of rewarding consumers for the detected defect. Choose which bonus method you like.	60% discount accrual;	
		35% accrual of bonuses, which can later be exchanged for goods;	
		5% service privileges (VIP cash desk, free delivery)	

Source: Authors' depiction.

## Results and discussions

For two decades, humanity has been actively discussing and solving the problem of packaging disposal. As a result, there are new technologies for packaging processing, new environmentally friendly packaging materials and packaging methods. However, there is another problem now that is more global and less studied. This problem is the rational use of natural resources. According to research by international organizations (UN, FAO and UNEP), the main source of environmental pollution in the world today is food waste. Every year the planet produces 4 billion tons of food, a third of which goes to waste (1.3 tons of waste) (14). In turn, the UN has developed a program “Save Food” which has a truly global goal, which is to find effective ways to reduce food waste and food losses, conservation and rational use of natural resources owned by humans, reducing human impact on the environment, and combating malnutrition and hunger worldwide. According to the Food and Agriculture Organization of the United Nations, the largest losses occur in four groups of consumer goods. Namely: meat and meat products, fish and seafood, vegetables and fruits, and dairy products. There are also significant losses at the points of sale and storage of food.

Within the framework of this initiative, the author proposed the creation of a concept of smart packaging (15), which would ensure proper storage of food and prevent food waste. One of the steps in implementing the concept of smart packaging is to develop a system for collecting and processing information. The system is implemented through hardware, software and includes social aspects of information relations. If the issue of technical support is clear, then the issue of information support, through the prism of a certain society, requires detailed research.

**Figure 4:**
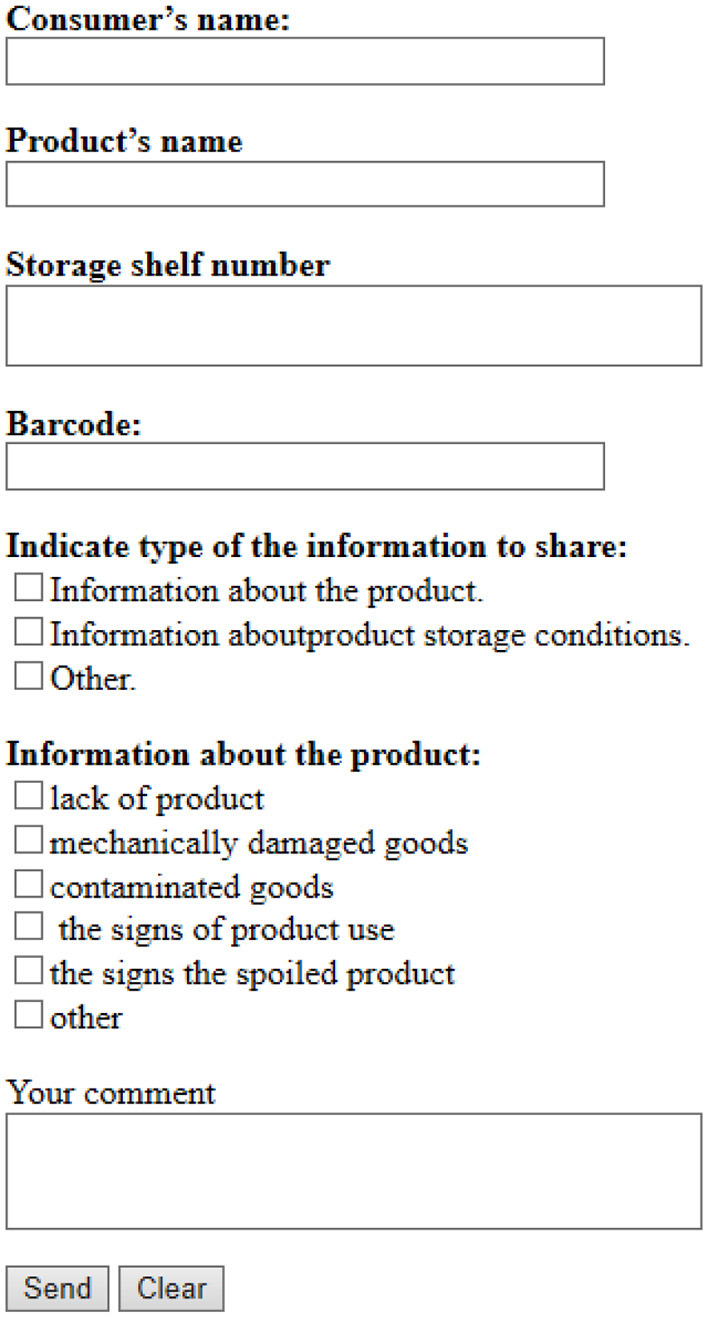
General view of the web form to be filled out by consumers. Source: Authors' depiction.

One of the elements of this system's functioning is the consumer. He/she is a source of information for the system and helps trade hall administrators prevent food waste. To identify and report illiquid goods, the consumer shall fill out a web form. The link to the web form is contained in the price tag in the form of a QR code. A sample web form is shown in Figure 4. By selecting the necessary items, the consumer informs the system about the availability of illiquid goods in a particular shopping area.

Each consumer can be characterized by a certain consumer behavior. Depending on the type of behavior, the consumer will take an active part in the system or not. In order to determine the level of consumer interest in participating in the system, a survey of potential participants was conducted. The author interviewed more than 1,000 potential consumers, who were asked 7 questions regarding the principles of creating a system to monitor product quality indicators. The results of the survey are shown in Table 1.

Analyzing the data obtained, it can be stated that the majority of respondents would like to participate in the system of monitoring the quality of goods. However, a large percentage of respondents indicate additional privileges (discount or bonus points). This is due to a certain type of consumer behavior. Therefore, in order to better understand how to encourage consumers to participate in the system best, it is necessary to consider the formation of consumer behavior and its types.

Returning to Figure 2, the formation of consumer behavior begins with thought for the rational one and with the feeling for the irrational one. If to analyze the rational behavior, the consumer wants to get the most benefits for the least resources. In the developed system of monitoring the quality of goods, the good has no material form and is an act of consumer action. In this case, it is filling out a web form. The action is voluntary and depends entirely on the desire or intention of the consumer. The resource is the personal time of the consumer that he spends on this action. Accordingly, in rational behavior, the consumer will seek a balance between the action and the time spent on it. In this case, a certain motivational lever may be an additional benefit, as a reward for resources spent. Such benefits can be discounts on certain groups of goods, discount cards, bonuses, which can later be exchanged for goods; or privileges in service.

For irrational behavior, the vector of attack is to evoke a certain feeling in the consumer, which will form an intention in the future. Analyzing the methods of influence, we shall outline the following:

- Imitation. Repetition of elements of environmental behavior plays a huge role in human life. This is a form of learning, socialization, and adaptation. Once in a new cultural environment, a person tries to imitate what the environment does. At the level of consumer behavior, imitation also plays an important role, although it is not caused by an attempt at self-defense from the unknown, but by intellectual laziness, abstention from choice, and so on. It is much easier and more comfortable to do “like others.”
- “Infection.” In contrast to imitation, it is a process that occurs in the emotional sphere, when “infected” in certain situations, a person catches the mood of the environment and acts accordingly. “Infection” is one of the oldest ways to integrate group activities and is characterized by spontaneity, as it occurs primarily in situations of large crowds—in stadiums, concert halls, carnivals, rallies and more. The mechanism of infection is more effective not at the level of an individual, but at the level of the group.
- Suggestion. From the point of view of studying consumer behavior, this is a specially organized type of communicative influence used in the media, fashion, advertising and others. The process of suggestion differs from imitation and emotional infection not only in content but also in mechanism of action. Suggestiveness is characterized, on the one hand, as a property of the psyche (uncritical perception of information), and on the other, as an active process of influencing a person. The lower the level of intelligence and the degree of critical thinking, the more a person is exposed to external influences.

In irrational behavior, it is possible to evoke the following feelings of consumers:

- “Participation and significance in a great cause.” The consumer understands that his actions can help the environment and reduce food waste.
- “I'm one of them.” The consumer joins the community with famous people, politicians who support this initiative.

To correct consumer behavior, the author proposes to use social engineering, namely the motivation for certain actions. These actions do not pose a threat and harm to the consumer and their implementation depends only on the wishes of the consumer. To understand the process of influence, it is necessary to consider the stages of information processing. Since the consumer's behavioral response to motivating factors depends on what psychological processes occur in the human mind, their consideration will help to understand and identify those factors that affect the likelihood of the stimulus passing through certain stages of information processing. Information processing is the process of obtaining, interpreting, storing, and reproducing a stimulus. According to William McGeer's model, it can be divided into five main stages.

**Figure 5:**
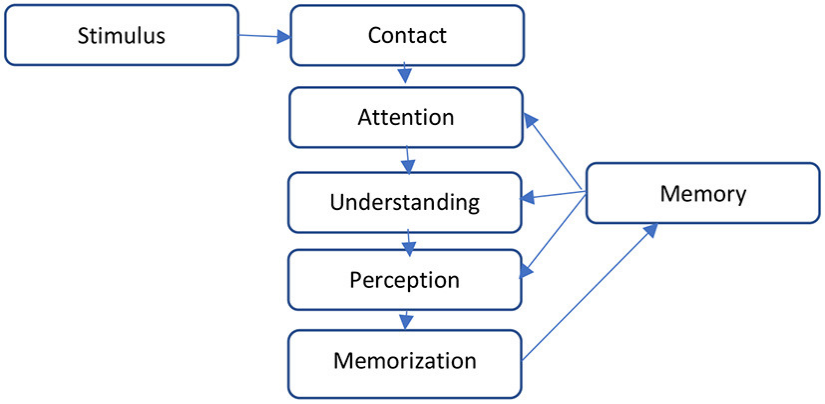
Stages of information processing according to the model of William McGeer.

Figure 5 shows that the stimulus, before entering the memory, must go through a number of stages of information processing: contact, attention, understanding, perception, and memorization. The effectiveness of communication, which will cause the appropriate behavioral response of the consumer, will depend on its ability to go through all these stages.

Information processing begins when the energy in the form of a stimulus reaches one of the five human senses. Contact occurs when physically approaching a stimulus that activates one or more sensations. When a person comes in contact with a strong enough stimulus, the sensory receptors are activated and the encoded information is transmitted to the brain through nerve endings. This phenomenon is a feeling. The task of social engineering is to evoke the right feeling with the help of special messages. Messages can have different emotional colors. For example, for rational behavior, they can be both neutral and positively colored with an emphasis on additional benefits. Both positive and negative messages can be used for irrational behavior. The purpose of the message is to awaken the feelings that will motivate the consumer to action. The tool of influence is the acting stimulus (message). The stimulus, in this case, may be a certain sound if it is an audio message, or a certain color, such as red, if it is a video message.

An interesting characteristic is the intensity of the stimulus. According to Weber-Fechner's law, the intensity of sensation is directly proportional to the logarithm of the stimulus force. It is believed that stimuli, the intensity of which is below the absolute threshold, can also affect the consumer, this is the so-called concept of subconscious belief. Therefore, at the stage of contact, the main task is to attract the attention of the consumer. Understanding is the third stage of information processing related to the interpretation of a stimulus. Understanding is influenced by the following factors: the level of knowledge, motivation or interest of the consumer.

An important stage in the information processing by the consumer is perception. In contrast to the process of sensation, in the perception of information a person does not percept the individual properties of objects and phenomena, but objects and phenomena of the world as a whole, that is, perception is holistic. This can lead to inadequate perception, distortion of the perceiving object or visual image, to the appearance of illusions of vision. Therefore, the wording in the message (stimulus) must be clear and understandable. Perception forms reactions of a cognitive or emotional nature.

Memorization is the last stage of information processing, where the interpretation and arguments are transferred to long-term memory.

Between types of behavior based on predominantly rational or irrational decision-making mechanisms, there is a wide range of transitional behaviors. Since consumer choice is not a one-time phenomenon, it is a process, its implementation very often reveals the involved rational and irrational mental mechanisms. The author approaches this problem pragmatically: the process of choice is divided into phases and each of them is considered separately as rational or irrational.

Given the impact of consumer behavior, the author has developed strategies to encourage consumers to fill out web forms. The strategy for rational consumer behavior is based on the creation of messages (stimuli) that will describe the additional or bonus opportunities, which the consumer will receive by performing a certain action.

The strategy for irrational consumer behavior is based on the formation of certain feelings in the consumer that will motivate him to take action.

An important point is to determine the phase of consumer choice when these strategies must be applied. To determine the phases, the author used the classical scheme in social engineering and adapted it to the research objectives.

**Figure 6:**
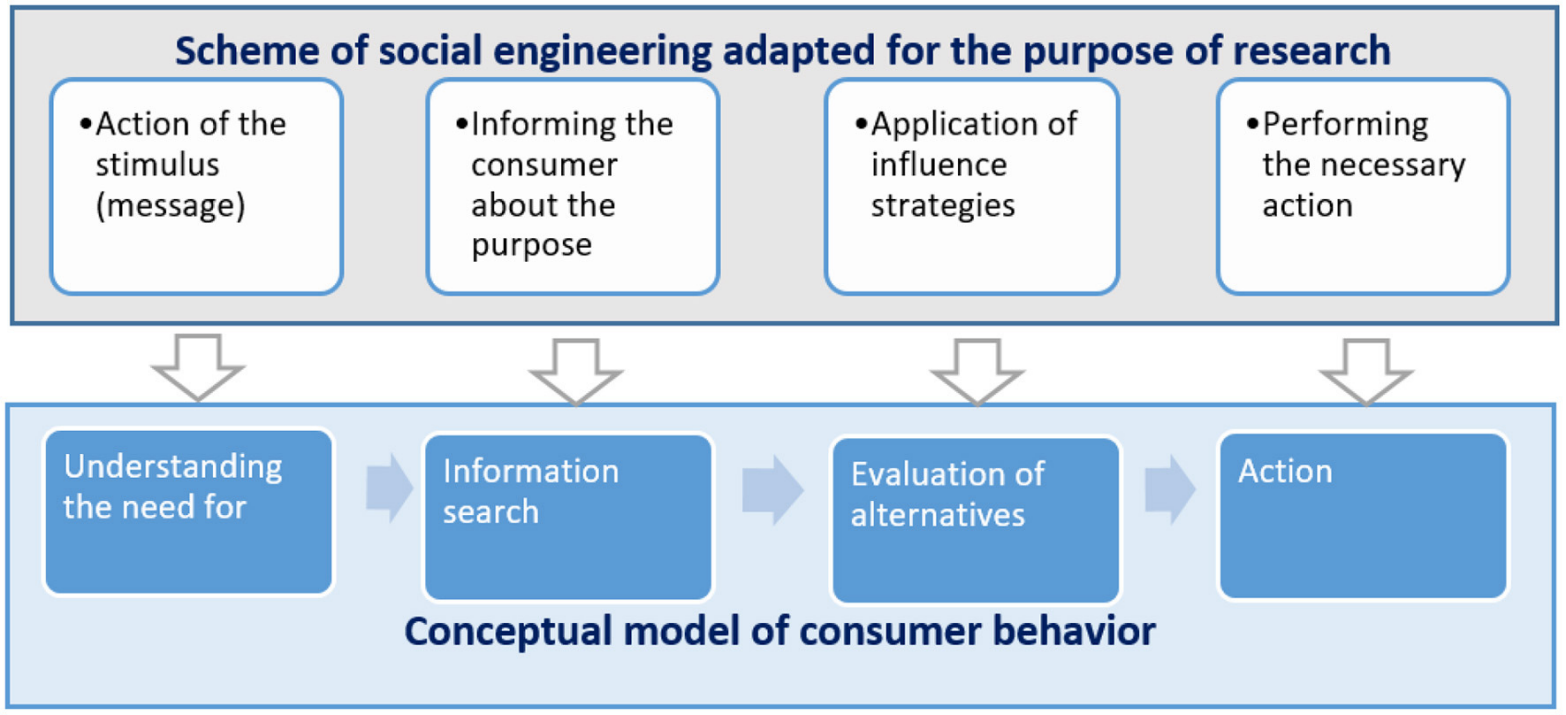
Scheme of influence on consumer behavior by methods of social engineering. Source: Researchers' elaboration based on research data.

The purpose of social engineering is to obtain information, but the paths to this goal are different. In the scheme of Figure 6 at stage 1, there is the formulation of the purpose of the impact on the object, and the question of the appropriateness of these actions is solved. The purpose of influence in the classical scheme of social engineering is always secret for the object of influence. In Figure 6, the object of influence is informed in advance about the purpose of collecting information. Informing the object can be done with the help of video and audio messages already in the trading hall, or as a preliminary mailing to a mobile phone or mobile application.

The second step is to identify the object and gather information about it. If he is a regular customer, he usually has a discount card and a purchase history. Typically, such consumers find it easier to join retailer initiatives.

The next step is to identify the vector of attack, or select a strategy to change consumer behavior. At this stage, it is required to determine the type and content of the message (stimulus). Accordingly, the next step is to apply this message. If it is a regular customer of the store, the stimulus can be sent to a mobile phone or mobile application of the store. For the casual consumer, it is possible to broadcast a message (stimulus) in the trading floor. You can use various means to motivate the object for more active participation, such as additional benefits, accrual of bonuses, promotional offers, and loyalty programs. These tools give the object a sense of completion and reward. Thus, the next stage of the scheme is performed by the object voluntarily and without coercion.

If the stimulus caused the required action and the consumer filled out a web form, you can assume that the strategy worked.

The author tested the developed strategies at the enterprise with the current grocery store. In particular, ICity LLC provided retail space and its own staff to test strategies. 500 consumers took part in the experiment. The experiment was conducted in two stages. In the first stage (I), consumers were not informed about the experiment, but next to the food there were signs with the words “If you find a defect in the product, report it” and a QR code with a link to the web form. The second stage (II) lasted 4 weeks and was the longest. For convenience, the author divided the second stage into substages. At weeks 1 and 2, consumers were informed according to a strategy for irrational consumer behavior. The screens in the trading hall broadcast videos about the pollution of the environment with food waste and calls to change this situation. There were signs with QR codes next to food.

**Figure 7:**
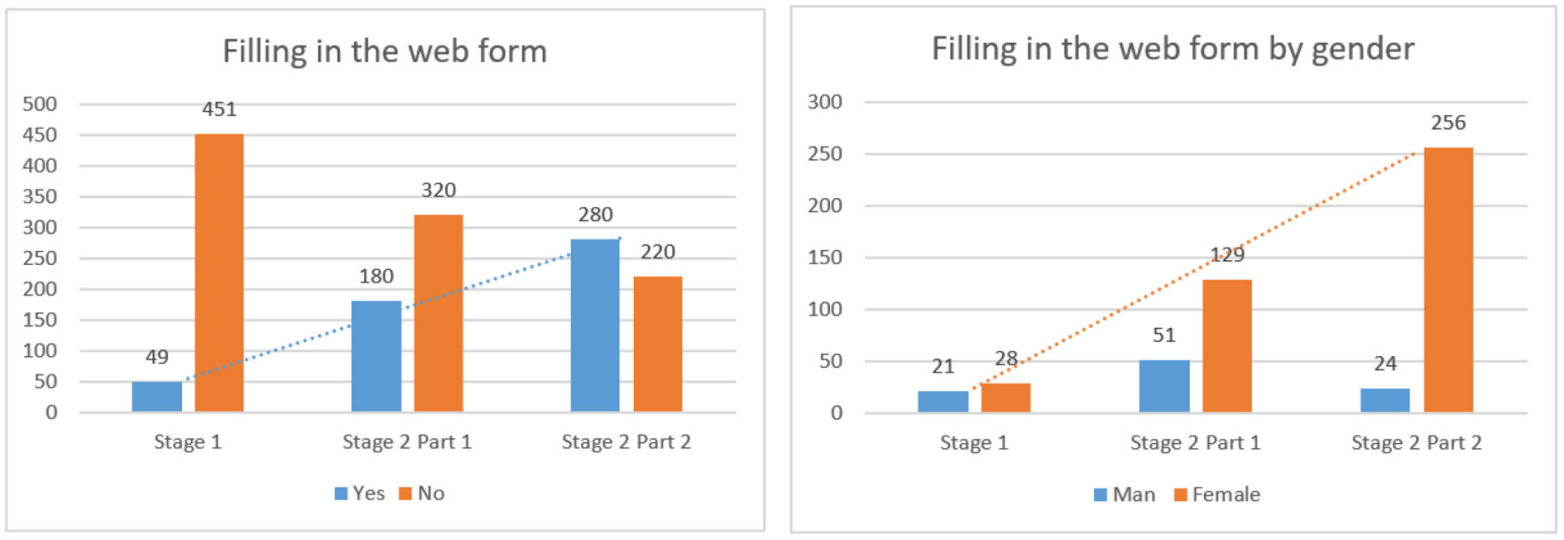
Results of the experiment. Source: Researchers' elaboration based on research data.

At weeks 3 and 4, a strategy for rational consumer behavior was applied to consumers. As in the previous 2 weeks, a database of consumers who have already filled out the web form was formed, it was possible to send a message (stimulus) to mobile phones. Also, at the entrance to the store, an audio message was broadcast about additional bonuses for filling out the web form. Signs were removed from the trade hall and only QR codes were left next to products. The results of the experiment are shown in Figure 7.

Analyzing the results of the experiment, we can conclude that the application of strategies increases significantly the active participation of consumers in the system of monitoring the quality of goods. Undoubtedly, there are participants in the experiment who did not take part despite the applied strategies. This is due to lack of time, lack of mobile phone at the time of the experiment or lack of interest in the problem. Conducting the experiment in three different stages precluded informing respondents about the final bonuses and benefits, which provided an opportunity to recreate the real environment of buyers. It can be argued that combining strategies for rational and irrational behavior increases the number of participants in the food quality monitoring system.

Also, analyzing the data from the chart, it can be argued that women are more inclined to adjust their consumer behavior. This is confirmed by the growth of the number of sent web forms from 28 at the first stage to 256 at the last stage of the experiment.

## Summary and concluding remarks

Social and economic research in the concept of smart packaging is a necessity. Without the participation of consumers, this concept cannot be considered complete. The system operates on the flows of information from the technical support and the social sphere. The combination of technical and social security provides a more complete and in-depth understanding of the information collection and processing system within the concept of smart packaging.

As a result of the experiment, new problematic issues in the study were revealed, namely the optimization of the web form interface and the consumer information system. A well-designed user-friendly interface will encourage the customer to inform the store administrators about these problems. We shall also consider the communication strategies used in the web framework interface:

– global strategy (i.e., be polite, be positive, be honest);

– local strategy (i.e., greetings, request for personal information, detailed description, gratitude for the client's help).

Of course, communication with the consumer is mandatory and transparent. It shall be noted that the client must have complete information about the procedure in which he participates. He must understand the importance of his feedback and must be motivated to provide as much information as possible to minimize food loss. At the same time, the process of gathering information shall not take too long, otherwise, the client will simply give up or reject the idea in the middle of gathering information while filling out the web form.

## Data availability statement

The original contributions presented in the study are included in the article/supplementary material, further inquiries can be directed to the corresponding author.

## Author contributions

The author confirms being the sole contributor of this work and has approved it for publication.

## Conflict of interest

The author declares that the research was conducted in the absence of any commercial or financial relationships that could be construed as a potential conflict of interest.

## Publisher's note

All claims expressed in this article are solely those of the authors and do not necessarily represent those of their affiliated organizations, or those of the publisher, the editors and the reviewers. Any product that may be evaluated in this article, or claim that may be made by its manufacturer, is not guaranteed or endorsed by the publisher.
